# Spinal Myxopapillary Ependymoma: A Rare Case and Review of Management Strategies

**DOI:** 10.7759/cureus.39381

**Published:** 2023-05-23

**Authors:** Cuauhtemoc Jeffrey Soto, Samuel D Novick, Avula Naga Laxmi Poojita, Saima Khan, Muhammad Waqas Khan, Shaniah S Holder

**Affiliations:** 1 Research and Development, Universidad Juarez del Estado de Durango, Durango, MEX; 2 General Surgery, Nassau University Medical Center, East Meadow, USA; 3 Medical Student, University of Nicosia Medical School, Nicosia, CYP; 4 Neurology, Kurnool Medical College Kurnool, Cuddapah, IND; 5 Internal Medicine, Sir Syed College of Medical Sciences for Girls, Karachi, PAK; 6 Medicine, Services Institute of Medical Sciences, Lahore, PAK; 7 Medicine, American University of Barbados School of Medicine, Bridgetown, BRB

**Keywords:** gross total resection (gtr), lumbar spine tumor, spinal mri, spinal ependymoma, myxopapillary ependymoma

## Abstract

Intramedullary myxopapillary ependymomas are rare spinal cord tumors primarily affecting young adults. Grade 2 tumors are associated with a higher proliferative index and potentially more aggressive behavior compared to grade 1 tumors. We present a case of a 30-year-old male who presented with a three-month history of progressive unilateral lower back pain that was refractory to analgesics. Neurological examination revealed bilateral lower limb weakness and sensory impairments in the L2 region. MRI confirmed a well-defined, enhancing intramedullary lesion at the L2 level, causing cord enlargement and edema. Diagnosis of grade 2 intramedullary myxopapillary ependymoma was made. Complete surgical resection was performed, confirming a grade 2 myxopapillary ependymoma. Postoperatively, the patient demonstrated significant improvement in lower limb function and sensation, with no tumor recurrence during long-term follow-up. Rehabilitation therapy was initiated, while close monitoring for complications and tumor progression was maintained. This case explores the etiology and features of intramedullary myxopapillary ependymomas and underscores the importance of early recognition, accurate diagnosis, and aggressive surgical management.

## Introduction

Ependymomas are rare neuroepithelial tumors derived from ependymal cells within the central nervous system [1]. They arise within the cerebral ventricles or the central canal of the spinal cord and affect approximately 0.29 to 0.6 per 100,000 people annually [1]. Spinal cord ependymomas account for 3-6% of cases and can be found at any level of the spinal cord, including the cervical, thoracic, and lumbar regions [2]. The risk factors associated with ependymoma formation remain poorly understood; however, certain lifestyle factors such as nutrition, body mass, and tobacco use may contribute to their development [3]. Childhood exposure to radiation, hereditary cancer syndromes like neurofibromatosis type two, and immunodeficient states such as post-transplantation or acquired immunodeficiency syndromes (AIDS) have been linked to an increased risk of spinal ependymoma formation [3]. 

According to the World Health Organization (WHO), ependymomas can be classified into four histopathological subtypes based on their grade of anaplasia. This aids in determining prognosis, and categories include (i) subependymoma, which is grade I, (ii) classic ependymoma, which is grade II, (iii) anaplastic ependymoma, which is grade III and associated with rapid growth and (iv) myxopapillary ependymoma (MPE), which was recently moved to the grade II category [4]. However, the site and histology of these tumors vary according to age, which may affect the prognosis. Classic ependymomas and subependymomas are more commonly observed in individuals aged 45 to over 65 years old, while anaplastic ependymomas are predominantly seen in children under 19 years old [5]. MPEs tend to occur in patients between 20 and 44 years old [5].

MPEs of the spine are extremely rare with an incidence of 0.01 per million people [6]. They predominantly affect the lumbosacral spine comprising 90% of all conus medullaris and cauda equina tumors [7]. Histologically, MPE tumor cells exhibit cuboidal or spindled shapes and secrete stromal mucin [8]. There are no specific risk factors associated with spinal MPE, and compression of the spinal nerve roots by the tumor leads to characteristic features such as radicular back and extremity pain, bladder and bowel dysfunction, as well as lower extremity weakness, sensory loss, and paresthesia if the lumbar spine is affected [9].

The optimal management of ependymomas poses a challenge for physicians; however, surgical resection is the most commonly employed treatment modality [10]. Surgical resection aims to remove as much cancerous tissue as possible to prevent recurrence and alleviate neurological symptoms in symptomatic patients [10]. The recurrence rate of ependymomas is approximately 50% and typically occurs within the first 13 to 25 months following the initial resection [11]. Recurrence is predominantly local, with metastasis occurring in 20% of cases [11]. Therefore, close follow-up to monitor for recurrence is crucial in preventing future complications. In this report, we present the case of a 30-year-old patient with complaints of back pain and extremity weakness who was found to have a spinal MPE.

## Case presentation

A 30-year-old Asian male, school teacher, presented to the outpatient neurology department of a tertiary care hospital with a three-month history of unilateral (left) lower back pain that was initially non-radiating and relieved by analgesics. Subsequently, for one month, the pain extended to the contralateral lower extremity, growing in intensity and becoming refractory to analgesic therapy. Furthermore, two weeks later, the patient presented with bilateral weakness in the lower limbs and sensory impairments in the L2 region involving light touch and pinprick sensations. He did not experience any urinary and fecal incontinence. His vital signs were normal. The patient's medical history was unremarkable, with no significant prior illnesses, weight loss, or current use of medications. He confirmed that he did not consume alcohol and was a non-smoker. Subsequently, he was admitted to the neurology ward for further assessment and the baseline investigations including complete blood count (CBC), liver function tests (LFT), renal function tests (RFT), electrolyte levels, and vitamin B12 assessment were carried out. Table 1 highlights these findings.

**Table 1:** Lab Parameters Hb: Hemoglobin, MCV: Mean Corpuscular Volume, WBC: White Blood Cell count, ALT: Alanine Transferase, AST: Aspartate Aminotransferase, BUN: Blood Urea Nitrogen, Cr: Creatinine, TSH: Thyroid Stimulating Hormone, INR: International Normalised Ratio, HbA1c%: Hemoglobin A1C, ESR: Erythrocyte Sedimentation Rate, CRP: C-Reactive Protein

Lab Parameters	Value	NORMAL RANGE
Hb (g/dL)	14.6	(13.5-17.5)
MCV (fl)	85.8	(80-100)
WBC (X10^9^/l)	6.1	(4.5-11)
Platelets (X10^3^/ul)	336	(150-400)
ALT (IU/L)	22	(7 to 55)
AST (IU/L)	30	(8 to 48)
BUN (mg/dL)	18	(6 to 24)
Cr (mg/dL)	0.8	(0.7 to 1.3)
Amylase (IU/L)	90	(30 to 110)
Lipase (IU/L)	75	(10 to 140)
TSH (mU/L)	2.7	(0.4 to 4.0)
INR	1	(1.1 or below)
HbA1c%	5.0	(Below 5.7%)
ESR	55	0 to 20 mm/hr
CRP	18	below 10 mg/L
B12	630	160-950 pg/ml

Upon a detailed neurological examination, the patient exhibited normal mental status and intact cranial nerve function. Motor examination revealed predominant motor weakness, with decreased strength noted in the hip flexors, knee extensors, ankle dorsiflexors, and toe extensors, graded as 4/5. His motor assessment suggested dysfunction in the descending corticospinal tract, indicating possible upper motor neuron involvement. Sensory examination revealed impaired sensation in light touch and pinprick testing, indicating decreased sensation to tactile stimuli, suggesting a disruption along the ascending spinothalamic tract. Deep tendon reflexes were slightly diminished, graded as 2/4, while Babinski reflex was present bilaterally. The Hoffmann test was negative, indicating no flexion or twitching of the thumb and adjacent fingers. The patient displayed a preserved anal reflex and there were no saddle paresthesia symptoms observed. Notably, the absence of saddle paresthesia, intact anal reflex, and negative Hoffman's sign suggested relative preservation of the sacral segments and associated nerve roots. Table 2 highlights these findings.

**Table 2:** Neurological Examination Findings

Category	Findings
Mental Status	Alert and oriented to person, time, and place Normal attention and concentration Intact memory (both short-term and long-term)
Cranial Nerves	Cranial nerves II-XII intact
Motor Examination (Muscle Strength Grading)	Upper Extremities: Shoulder abduction: Grade 5 Elbow flexion/extension: Grade 5 Wrist flexion/extension: Grade 5 Finger abduction/adduction: Grade 5
	Lower Extremities: Hip flexion/extension: Grade 4 Knee flexion/extension: Grade 4 Ankle dorsiflexion/plantar flexion: Grade 4 Toe dorsiflexion/plantar flexion: Grade 4
Sensory Examination	Diminished sensations to light touch and pinprick No saddle paresthesia
Reflexes	Deep Tendon Reflexes (DTRs) Grading: Biceps: Grade 2+ (Diminished) Triceps: Grade 2+ (Diminished Patellar: Grade 2+ (Diminished) Achilles: Grade 2+ (Diminished)
Superficial Reflexes	Plantar reflex: Bilateral positive Babinski
Coordination and Gait	Normal coordination and gait
Special Tests	Hoffmann test: Negative Anal reflex: Present

**Figure 1:**
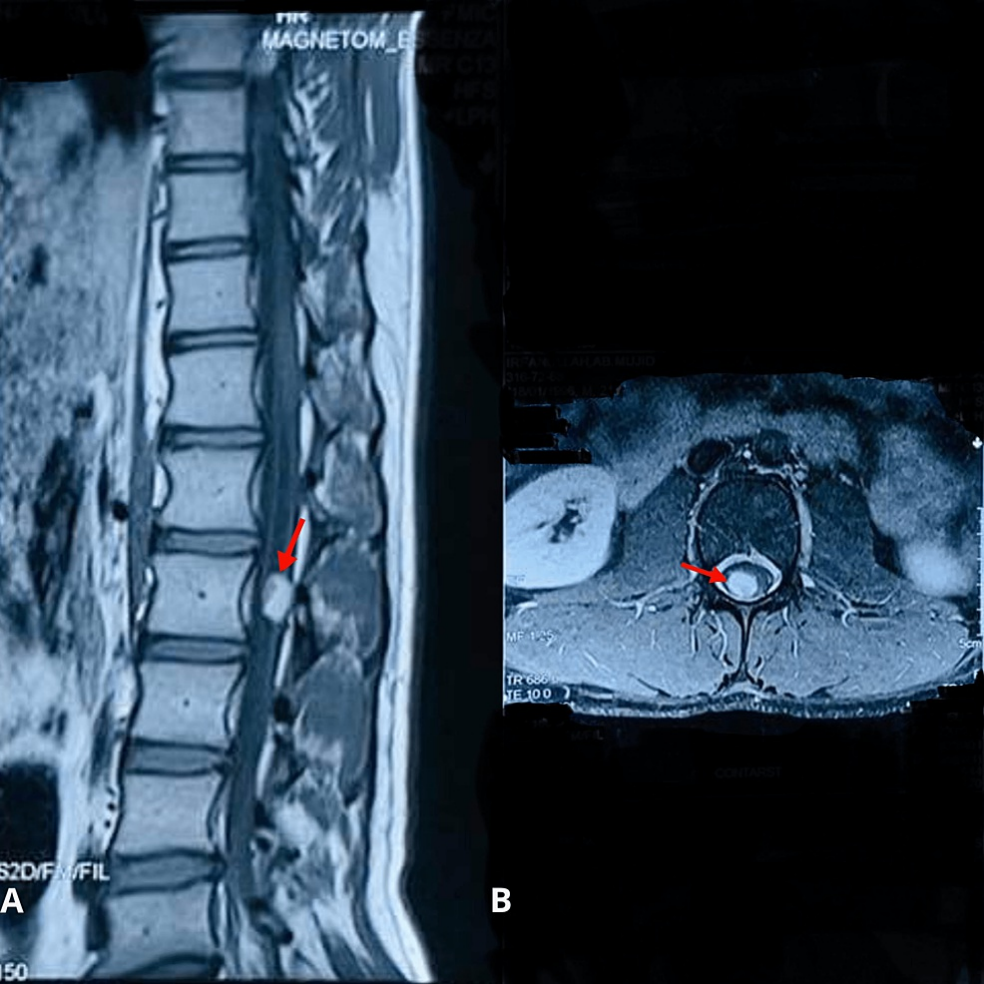
MRI of the spine with IV contrast showing a sagittal view (A) and axial view (B) of the lesion at level L2 (red arrows). IV: Intravenous

Based on the extensive assessment of the patient's neurological examination, which encompassed motor, sensory, and reflex functions, along with a thorough review of the patient's medical history, there was a strong suspicion of an intramedullary tumor at the L2 level. In order to confirm this diagnosis and to exclude alternative potential differentials, such as demyelinating disorders like multiple sclerosis, inflammatory conditions such as transverse myelitis, B12 deficiency, Guillain-Barré syndrome (GBS), and central cord syndrome, a spinal magnetic resonance imaging (MRI) was conducted. The MRI revealed a well-defined, enhancing intramedullary lesion at the L2 level, as well as cord enlargement and edema. The tumor exhibited hyperintensity on T2-weighted imaging and isointensity on T1-weighted imaging, with varying enhancement on contrast-enhanced imaging. This is highlighted in Figure 1.

The MRI also revealed evidence of compression of the spinal cord at L2. Based on his physical examination findings, clinical presentation, and confirmatory imaging with MRI, the diagnosis of an intramedullary ependymoma at the L2 level was established.

Following the diagnosis of an intramedullary ependymoma at the L2 level on MRI, a complete surgical resection was advised due to the lesion's limited dimensions. The surgery was a complete success with no intraoperative or immediate postoperative complications. Further microscopic examination of the specimen revealed the presence of neoplastic tissue organized in clusters and elongated processes. The neoplastic cells exhibited a cuboidal to elongated morphology and were conspicuously arranged around myxoid cores. Special staining with Periodic Acid-Schiff (PAS) with or without Alcian Blue (AB) demonstrated the presence of mucin within the myxoid cores. Immunohistochemical staining was performed, revealing positive reactivity for glial fibrillary acidic protein (GFAP) and focal positivity for epithelial membrane antigen (EMA). These findings are consistent with a diagnosis of intramedullary spinal myxopapillary ependymoma, grade II.

**Figure 2:**
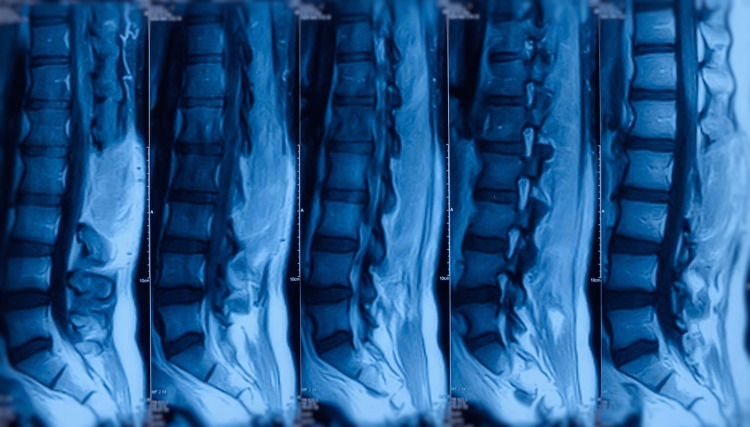
Postoperative spinal MRI with IV contrast IV: Intravenous

Following the surgery, there was a noteworthy improvement in the patient's lower limb strength and sensation, enabling him to walk independently. The patient was under close observation for any possible signs of neurological impairment or adverse outcomes. There were no observed instances of new neurological impairments. An MRI was conducted postoperatively to verify the complete removal of the tumor and to evaluate the presence of any remaining or recurring tumor. Figure 2 below shows the postoperative spinal MRI indicating complete tumor resection.

The patient also began a rehabilitation program to facilitate the recovery of both motor and sensory function as well as mitigate any potential complications such as deep vein thrombosis and pressure ulcers.

Sustained surveillance is imperative for individuals with spinal cord tumors to detect any possible relapse or advancement of pathology, so a long-term follow-up was carried out using MRI, which was routinely performed at fixed intervals, usually every six to 12 months for three years following the surgery, with less frequency after the first year. Additionally, it was recommended that he follow up with his neurologist and receive occupational therapy and psychological assistance in order to enhance his recuperation and overall well-being.

## Discussion

The underlying etiology of ependymoma development is not well known. These tumors are heterogeneous, exhibiting various subtypes and variations in cellular and clinical characteristics [12]. Molecular classification of spinal ependymomas includes (i) N-MYC proto-oncogene amplified, (ii) N-MYC proto-oncogene nonamplified, (iii) spinal ependymoma not otherwise specified (NOS), and (iv) myxopapillary ependymoma, which was the specific subtype in this case [12]. MPEs of the spine account for only 1 to 5% of all spinal neoplasms and 13% of all spinal ependymomas [7].

Symptoms commonly associated with MPEs include lower back pain and lumbosacral radiculopathy due to compression of nerve roots [13]. In this case, the patient presented with severe neurological symptoms, including decreased sensation below the L2 dermatomal distribution and weakness in hip flexion, knee extension, ankle dorsiflexion, and toe extension. Hip flexion is controlled by the L2 segment via the iliopsoas muscle, knee extension involves the quadricep muscles coordinated by L3, while ankle dorsiflexion and great toe extension are governed by L4 and L5 through the tibialis anterior and extensor hallucis muscles [14]. The absence of weakness in hip abduction or knee flexion suggests partial involvement of L5 [14]. These findings indicate that although the tumor was located at the L2 level, surrounding edema and cord expansion resulted in extensive compression of nerve roots, leading to these physical manifestations. Other symptoms associated with MPEs include bowel and bladder dysfunction, which occurs in 30% of patients, as well as cauda equina syndrome characterized by limb dysfunction, saddle anesthesia, and bladder and bowel dysfunction; however, the patient in this case did not exhibit these symptoms [12]. Tumor growth increases the risk of hemorrhage due to vascular degeneration, potentially resulting in permanent neurological deficits [13]. Early diagnosis and treatment are crucial in preventing such severe complications.

Thorough neurological evaluation and the use of imaging tests aid in the diagnosis of spinal ependymomas [9]. Spinal MRI is the preferred diagnostic imaging modality for MPEs as it allows visualization of the tumor to assess its size and relationship with surrounding intraspinal structures [9]. MPEs typically appear isointense or hypointense on T1-weighted imaging and hyperintense on T2-weighted imaging due to their mucinous content [7]. Additional MRI findings suggestive of MPEs include caudal degeneration, present in 50% of patients, tumor extension involving multiple lumbosacral nerve roots, and signal inhomogeneity characterized by a region of the tumor margin exhibiting lower intensity on T2-weighted imaging [7]. In this particular case, the tumor was small, with well-defined borders, and showed isointensity on T1-weighted imaging and hyperintensity on T2-weighted imaging. Surgical excision is considered the gold standard treatment for spinal cord ependymomas, particularly for MPEs [15]. Gross total resection (GTR) aims to completely remove the tumor while preserving healthy spinal tissue and is associated with complete resolution of symptoms and lower rates of recurrence [15]. Studies suggest that well-encapsulated MPEs have a higher success rate of being resected without severe neurological complications [9,16]. However, postoperative neurological deficits, such as permanent bladder and bowel dysfunction and sensory loss, may occur if the tumor's capsule is weak and allows infiltration and penetration by the surrounding nerve roots [9,17]. In such cases, subtotal resection followed by adjuvant radiotherapy may be necessary [9]. The use of radiotherapy and its benefits in preventing recurrence are still subject to debate. A cohort study by Lee et al. examined functional outcomes in patients who underwent total resection versus subtotal resection with the addition of radiotherapy [17]. Their findings indicated that postoperative radiotherapy after partial resection did not correlate with a longer time to recurrence and that GTR of the tumor was associated with an improved prognosis [17].

MPEs have a high rate of local recurrence, with reports of post-operative recurrence ranging from one to eight years [18]. Marchesini et al. studied the long-term outcomes of 125 patients who underwent surgical treatment for spinal ependymomas and found that although histopathological subtype did not directly correlate with the recurrence rate, some features commonly found in MPEs were identified [16]. Histologically, MPEs are characterized by papillary structures resulting from perivascular degeneration [7]. The tumor cells are cuboidal or spindle shaped, secrete stromal mucin, and surround blood vessels within the center of the papillae, which may undergo vascular hyalinization [8]. Other histological features of MPEs include perivascular pseudorosettes, mucin-rich microcysts, and mitotic figures [7]. In this case, the cells were cuboidal and arranged around a myxoid core containing mucin. Because of these features, the WHO updated its classification of central nervous system (CNS) tumors in 2021, elevating MPEs from grade I to grade II, reflecting the increased recurrence risk [12]. Recurrence is attributed to the tumor's tendency to infiltrate the conus medullaris and spinal cord parenchyma [19]. A report by Sonneland et al. involving 77 patients with myxopapillary ependymoma who underwent surgical resection demonstrated a recurrence rate of 10% in cases of complete tumor resection and 19% in cases of subtotal resection [20]. This study highlights the importance of radical initial resection and sustained postoperative surveillance with imaging.

In the presented case, the patient had a small intramedullary ependymoma, and his symptomatic presentation, diagnostic imaging findings, and histopathological features all pointed to MPE. Spinal MRI confirmed the diagnosis and complete gross total resection and subsequent physical rehabilitation led to the complete resolution of his neurological symptoms. Close follow-up with MRI at regular intervals proved effective in ensuring the absence of tumor recurrence.

## Conclusions

Spinal myxopapillary ependymoma is a rare grade II CNS tumor characterized by perivascular degeneration and stromal mucin secretion. It is heterogeneous in nature and has a variety of clinical presentations including lumbosacral radiculopathy, bladder and bowel dysfunction, and cauda equina syndrome. Swift and appropriate diagnostic and therapeutic management are crucial to prevent permanent neurological consequences. Physicians should consider conducting a spinal MRI as the gold standard diagnostic tool when presented with symptoms associated with MPEs. It is worth noting that small, well-encapsulated tumors with mild preoperative neurological features are associated with a better prognosis and improved functional outcomes. Given the elevated recurrence rate of MPEs, strict post-resection surveillance with spinal MRI may be beneficial. Post-operative spinal MRI screenings can facilitate early detection and treatment of relapsed MPEs. Further research is needed to investigate the long-term benefits of MRI surveillance and its impact on patients' quality of life.
